# Using archaeometabolomics to characterise tobacco-usage in human cortical bone from 17^th^ to 19^th^ century Dutch communities

**DOI:** 10.1371/journal.pone.0361147

**Published:** 2026-10-30

**Authors:** Andrea Bonicelli, Anna M. Davies-Barrett, Maria Serrano Ruber, Jatinderpal Sandhu, Donald J. L. Jones, Sarah A. Inskip

**Affiliations:** 1 School of Heritage and Culture, University of Leicester, Leicester, United Kingdom; 2 Research Centre for Field Archaeology and Forensic Taphonomy, School of Law and Policing, University of Lancashire, Preston, United Kingdom; 3 School of Archaeology, University College Dublin, Belfield, Dublin 4, Ireland; 4 Leicester Cancer Research Centre, RKCSB, University of Leicester, Leicester, United Kingdom; 5 The Leicester van Geest MultiOmics Facility, University of Leicester, Leicester, United Kingdom; Vilnius University: Vilniaus Universitetas, LITHUANIA

## Abstract

Detecting tobacco consumption in historical populations is a complex challenge often encountered by archaeologists. Traditional methods typically involve the morphological examination of teeth for pipe-notches and/or staining. However, when teeth are absent, discerning tobacco-usage becomes impossible, highlighting the need for alternative approaches. Recently, metabolomics has gained attention as a promising tool in the analysis of archaeological artefacts and human remains, offering new insights into ancient smoking habits. In this study, we applied a combination of reversed-phase (RP) C18 and hydrophilic interaction liquid chromatography (HILIC), both coupled with mass spectrometry, to analyse a Dutch population spanning pre- and post-tobacco archaeological contexts. Our statistical approach focused on the multivariate analysis of each assay, followed by the integration of the two datasets, and Random Forest modelling to test the effect of covariables. The results demonstrated some separation between tobacco consuming and non-consuming groups, as evidenced by distinct metabolomic profiles in the omic blocks. The single block model exhibited a bias towards correctly classifying the tobacco-consumer group, with a balanced estimation error of 0.375 for the C18 assay. Additionally, integrating the two datasets produced a similar classification accuracy potentially driven by the RP classification potential, particularly when distinguishing between pre- and post-tobacco contexts. Overall, our findings confirm the potential of mass spectrometry-based metabolomics as a powerful tool for studying tobacco consumption habits in past populations, although further work is needed to disentangle wider affects from other variables and tightly dated samples are needed. This method could be validated in the future and complement current morphological methods.

## Introduction

Tobacco consumption has a long human history that spans over 12,000 years [1]. However, there have been few attempts to examine how the modern form of tobacco consumption that emerged in the sixteenth century relates to age, sex and gender, and how it has influenced health in the *longue durée*. This knowledge gap is due to a lack of historical data on tobacco-use practices, preventing us from linking its adoption to changes in historic disease patterns. Bioarchaeological research (the analysis of archaeological human remains), which has relied on identifying tobacco consumption via the macroscopic observation of pipe-notches – dental abrasion caused by the stem of a clay pipe in the teeth – and dental staining, only produces a partial picture of consumption (e.g., those that used clay pipes) [2] and as such additional biomolecular based methods are required to assess tobacco use on a population level.

Metabolomics is the systematic and statistical profiling of small molecules (<1.5 kDa) in a biological sample, which can be at a cellular level, through to a whole organism. Metabolomic workflows aim to study the processes of metabolism through the characterisation of small molecules (metabolites) in biological samples, which act as a snapshot of physiological state at the time of analysis. This makes it a powerful tool for the analysis of different types of phenotype, including of relevance here, intoxicant use [3]. Metabolomics is broadly approached in two ways, a targeted approach, where a predefined set of metabolites are explored, or in an untargeted or discovery manner, where all possible small molecules are examined for investigative purposes or characterisation (e.g., looking for biomarkers or differences between groups). The complete set of metabolites in a biological sample, is known as the metabolome.

There is a long history of studying small molecules from archaeological contexts or materials and this has been strongly focused on residues recovered from material culture, especially consumables but have also included sealants, inks, fabrics and human dental calculus [4]. There have been targeted studies, focusing on specific types of molecules, or untargeted studies that have necessarily limited sample sizes due to the studies reliance on archaeological material [5–10]. However, there have been few large-scale untargeted approaches, and very few studies that have involved archaeological (human) bone until recently [7], [11]. In addition, some studies have shown it is possible to recover metabolites relating to tobacco consumption from pipes, other material culture and historical plants [5], [10], [12], [13] as well as human dental calculus (mineralised plaque [14]).

Recent research by our group was one of the first to undertake large-scale untargeted metabolomics approaches on human skeletal remains, demonstrating that it was possible to recover and meaningfully statistically analyse metabolites from archaeological bone [15]. As part of the Tobacco, Health and History project, which aims to look at the impact of tobacco on health prior to the 20^th^ century, we developed a novel metabolomic approach for identifying tobacco-consumption status in English archaeological human remains, offering a first glimpse of tobacco consumption rates in two early-modern English archaeological populations [4]. This study found differences in the cortical bone metabolic profiles of 323 archaeological tobacco consumers (likely smokers) and non-consumers. These differences were significant, allowing for the assignment of tobacco use status (consumer or non-consumer) to individuals of unknown tobacco-use history. The aim of this research is to assess whether a similar approach can be applied to an independent Dutch population, further validating the approach when applied to other geographical regions and socially distinct populations. In addition, we wanted to determine whether using additional data from HILIC analysis can improve the tobacco use assignment accuracy. We aim to further test whether this is a valid approach for the detection of archaeological phenotypes.

Our hypothesis for this research is that the metabolomic bone phenotype will differ between those with macroscopic evidence of tobacco consumption, and those that pre-date tobacco, or had no visible evidence of tobacco use. These differences would be distinct enough that we could assign tobacco consumption status to individuals where we did not know whether they used tobacco or not. Overall, this would provide information that would allow us to better understand the tobacco consumption in early modern Dutch populations prior to the emergence of the modern cigarette.

To achieve this, we assessed individuals with known tobacco histories to create models with which we could then use to classify individuals with unknown tobacco-use histories, with a view to reconstructing tobacco consumption patterns in three Dutch archaeological groups. Using a multi-solvent extraction method and LC-HRMS/MS (C18 and HILIC), we obtained molecular feature data for all 257 samples using C18, and 208 of the same samples using HILIC. Based on the data from the individuals with known tobacco histories, we created individual models using C18 and HILIC data separately and combined using multivariate testing. The results showed that there was a degree of separation between the known tobacco consumers and pre-tobacco individuals for both C18 and HILIC data, thus supporting the hypothesis. Combination of C18 and HILIC data into one model slightly increased the accuracy of identification for the 79 overlapping samples with no evidence for tobacco-use. To evaluate the extent to which demographic and archaeological covariates might confound smoking-related metabolomic signatures, we implemented a set of sensitivity analyses combining variable importance assessment, feature residualisation, and covariate-adjusted classification. Feature residualisation was performed using a non-linear random forest regression model. Using both models, we were able to assign tobacco consumption status to 95 individuals with no macroscopic evidence for tobacco-use. The results support the findings from our previous paper [4] that metabolomics can be used to distinguish between consumption phenotypes in past populations. However, far more work is needed to validate these molecules before they can be used as targeted biomarkers.

Using the metabolomic data, we are now able to show that, within the groups assessed, tobacco consumption was strongly associated with men, with only one ‘non-consuming’ male identified at two of the post-tobacco sites. Far fewer women presented as tobacco consumers, but more than might be assumed based on narratives drawn from historical sources [16]. Like our previous research, the work presented here shows that archaeometabolomics is a powerful tool for reconstructing the impact of cultural consumption behaviours in the past and across different population groups, and it also has significant potential for looking at other phenotypes which are invisible from the skeleton macroscopically.

## Materials and methods

### Sample composition and sampling strategy

As the aim of this research was to assess whether it was possible to identify differences in the metabolomic phenotype of tobacco consumers and non-consumers in a Dutch population as per our previous study, we sampled bone from 257 Dutch individuals that dated before and after the introduction of tobacco (see Table 1 and S1 Table). This sample consisted of individuals dating to c. AD 1650–1870 known to have been smokers from the presence of pipe-notches and lingual staining (positive controls); individuals from the same time period with no evidence of tobacco consumption; individuals dating to c. AD 1350–1650, the majority of which likely pre-dated the popularisation of tobacco consumption, and especially pipe smoking, in the Netherlands, which was likely around the early 17^th^ century (negative controls; referred to here as ‘pre-tobacco’ group) [16–18] (see Table 1, and S1 Text for site information); and individuals where tobacco consumption could not be ascertained macroscopically, usually due to a lack of preserved teeth.

**Table 1:** Sample composition by chromatographic dataset and archaeological period.

Dataset	Period	N	Absent	Present	% Present
C18	Medieval (pre-tobacco)	59	51	8	13.6%
C18	Post-medieval (post-tobacco)	207	35	172	83.1%
HILIC	Medieval (pre-tobacco)	45	39	6	13.3%
HILIC	Post-medieval (post-tobacco)	152	26	126	82.9%

As an additional key area of research on past tobacco use is to assess how consumption practices relate to sex and gender, social status and lifestyle, as health inequalities can often be tied to particular social identities, Dutch individuals were sampled from population groups representing men and women from rural, and upper-middle and lower-middle class urban communities. A total of 50 individuals were sampled from the church site of Broerenkerk in the city of Zwolle (ZW), in the Dutch province of Overijssel. The burials from Zwolle date from between c. AD1675 and 1828 with almost all of those included within the sample falling between AD1819 and 1828 (identifiable via burial records). The sample consists of well-to-do middle-class individuals from the city, who were affluent enough to be buried in the desirable position under the floor of the church [19]. A total of 107 individuals from the church cemetery of Eusebiuskerk in the city of Arnhem (AR), province of Gelderland, were sampled. This cemetery can be roughly divided into two groups based on approximate burial date: c. AD1350–1650, from which 48 individuals were sampled, the majority likely to predate the introduction of tobacco to the Netherlands, and c. AD1650–1829, from which 59 individuals were sampled who all lived in the period when smoking was popular. In contrast to Zwolle, individuals from the churchyard of Eusebiuskerk are tentatively concluded to have been of average or low socio-economic status [20,21]. Finally, a further 100 individuals were sampled from a church cemetery population from Keyserkerk in Middenbeemster (MB). Burials from this cemetery date from approximately AD1638–1866, 38 of which known to date to between AD1829 and 1866 due to burial records [22]. This cemetery served the entire municipality of Beemster (72 km^2^ region), a rural community mainly consisting of dairy farmers and artisans [22–26].

Based on the sampling method outlined by Badillo-Sanchez and colleagues [4,15], individuals were selectively sampled for the presence of preserved femora and for a relatively equal distribution of males, females, and different adult age groups (no juveniles were sampled as part of this study in order to limit the number of variables that could influence the metabolome). Age and sex were estimated according to standard osteological practice [27–35]. Individuals were allocated into male, female, or unknown sex groups, and into the age categories of young adult (20–34 years), middle adult (35–49 years), mature adult (50 + years), or adult of unknown age (20 + years). Tobacco-consumption status was macroscopically evaluated using the methods outlined by Davies-Barrett and Inskip [36], which requires the analysis of the dentition for evidence of tobacco staining or dental enamel abrasion related to the practice of smoking a clay pipe. Individuals were allocated into the following tobacco-consumption categories: tobacco consumer (evidence for dental staining and/or pipe abrasion), non-tobacco consumer (no macroscopic evidence for tobacco consumption), or unknown tobacco consumption status (dentition too incomplete to evaluate).

Approximately 75 mg of cortical bone was sampled from one femoral midshaft of each individual, according to methods outlined by Badillo-Sanchez et al. [4,15].

### Metabolite extraction material

Water Optima LC/MS Grade, and Methanol Optima LC/MS Grade were obtained from Thermo Scientific (Hemel Hempstead, United Kingdom). Ethanol hypergrade for LC-MS LiChrosolv was acquired from Merck (Rahway, NJ, USA) All chemicals were chilled on ice before extraction.

### Metabolite extraction

Metabolite extraction was carried out based on the Badillo-Sanchez et al. [4,15] protocol. In brief, 50 mg aliquots of the bone samples were placed in new 1.5 mL plastic tubes with O-ring caps and six 3 mm diameter ceramic beads (ZrO_2_). Two hundred µL of cold methanol were added to each tube. The tubes were vortexed for 10 seconds and then homogenised in a BeadBlaster 24 Microtube homogenizer (Benchmark Scientific Inc., USA) for six cycles of 20 seconds of homogenization followed by 25 seconds of pause. After homogenization, the tubes were centrifuged for 5 minutes at 9500 RCF. The supernatant was then removed and stored separately. Next, 200 µL of ethanol were added to the initial tubes containing the precipitate and ceramic beads, and the homogenization and centrifugation steps were repeated. The resulting supernatants were combined. This was followed by the addition of 200 µL of water to the initial tubes, repeating the homogenization and centrifugation steps once more. The resultant supernatants were also combined. The combined alcohol-water solution was divided into two halves, labelled Extract A and Extract B. Extract A was filtered through a pre-rinsed Microcon-10 kDa centrifugal filter unit using a centrifuge for 30 minutes at 14,000 RCF. Meanwhile, 200 µL of water were added again to the initial tubes with the precipitate and ceramic beads, repeating the homogenization and centrifugation steps. The supernatant from this step was divided, with one half added to Extract A and filtered again as described, and the other half added to Extract B. Both Extract A and Extract B samples were then centrifuged and filtered through a 0.22 μm membrane (Corning Costar Spin-X). The resulting filtered extracts were placed in a speed vacuum for 2 hours and subsequently frozen with liquid nitrogen for overnight freeze-drying. The dried extracts were stored at −80 °C. To ensure the process quality and data integrity, extraction blank samples were prepared simultaneously on each batch of extractions. Samples were then randomised prior to analysis.

### Liquid chromatography mass spectrometry

Untargeted analyses were carried out using a high-flow Acquity UPLC system coupled with a Waters Synapt G2 HDMS system (Waters Corporation, Manchester), operating in profile mode (full scan) across the m/z 50–1500 range in positive ionisation mode using mobility MS1 acquisition. The capillary voltage was set at 3.00 kV, the sampling cone at 30 V, with a source temperature of 120 °C. The desolvation temperature was 600 °C, and the desolvation gas flow was 1000 L/h. Both the IM and TOF cells were pre-calibrated, and leucine enkephalin (lock mass m/z 556.2771) was used as a lock spray, infused at 10 µL/min. Chromatographic separations were conducted using a reversed-phase (RP) C18 column (Waters Acquity UPLC BEH C18, 2.1 × 100 mm, 1.7 μm). Samples were stored at 4 °C in the autosampler, with a 5 µL injection volume per run. The flow rate was maintained at 0.4 mL/min, with the column temperature set at 40 ± 2 °C. Separation was achieved using 0.1% formic acid in water as mobile phase A and 0.1% formic acid in acetonitrile as mobile phase B. The RP gradient elution program began with 2% B, held for one minute, followed by a linear increase to 20% at minute 5, 25% at minute 6, 75% at minute 7, and 98% at minute 7.5, maintaining 98% B until minute 8, then reversion [4,15].

For chromatographic separations using hydrophilic interaction liquid chromatography (HILIC), an Acquity UPLC BEH HILIC column (2.1 × 100 mm, 1.7 μm) was employed. Samples were kept at 4 °C in the autosampler with a 5 µL injection volume. The flow rate was 0.4 mL/min, and the column temperature was maintained at 40 ± 2 °C. The solvents used were 0.1% formic acid in water (mobile phase A) and 0.1% formic acid in acetonitrile (mobile phase B). The HILIC gradient elution program started with 3% A for the first minute, gradually increasing to 20% over the next three minutes, then to 25% within one minute, 75% over the following two minutes, and 97% in the next minute. This was maintained for half a minute before returning to the initial conditions of 97% B over 1.5 minutes, followed by a four-minute equilibration (Badillo-Sanchez et al., 2024).

### Extract blanks and quality control (QC) and quality assurance (QA)

Quality assurance (QA) procedures were implemented throughout the workflow to minimise variation in the extraction and analytical phases and assure the quality of the metabolomic study [37]. Four to six injections of pure solvent (injection blank sample) were performed at the beginning of each sequence, followed by the injection of extraction blank samples three times per sequence. Pooled quality control (QC) samples, prepared by mixing 5 µL of each sample extract after reconstitution for each chromatographic technique, were injected six times at the beginning of each chromatographic sequence following the instrumental blank samples to further condition and stabilise the chromatographic column. Additionally, QC samples were injected after every 6–8 randomised samples to monitor analytical platform stability and after each extraction blank injection to maintain MS instrument stability. To prevent instrumental and statistical bias, all biological samples were randomised prior to injection. To test technical variability and potential carryover during the injection of different sequences, a quality control standard mixture (LC–MS QC STD Part number 186006963−1, Waters) was injected at the beginning and end of each sequence, with retention times and ions evaluated. Injection order is reported in metadata at https://github.com/SarahAInskip/THH-Dutch-2026

### Data pre-processing

Pre-processing was performed using Progenesis QI software (Nonlinear Dynamics). For reversed-phase chromatography, experimental runs were aligned, and peak picking was conducted within the 1–8-minute range, while for HILIC chromatography, this was done between 1 and 7.5 minutes. Normalised abundances were utilised for subsequent analyses. Adducts for the C18 column were selected based on Badillo-Sanchez et al. [4]. including: M + H, 2M + 3H_2_O + 2H, M + ACN + H, M + H-H_2_O, M + 2Na-H, 2M + H, M + Na, M + H-2H_2_O, M + ACN + Na, 2M + ACN + H, M + 2H + Na, M + 2H, M + 2Na, 2M + ACN + Na, and M + 2Na + H. For HILIC chromatography, the selected adducts were: M + H, M + Na, M + K, M + H-H_2_O, and M + 2H.

### Compound annotation

Putative annotation of the features was conducted using the data identification window of the Progenesis QI software. Data comparisons were made considering all potential matches across the following libraries:

1. METLIN MS/MS library 2017 for Progenesis QI plugin with a tolerance of 3-ppm for both precursor and fragment, and elemental constraints set to H = 0–150; C = 0–100; N = 0–10; O = 0–30; F = 0–4; Mg, P, S, Cl, Br, I = 0–2.
2. LipidBlast library plugin with a 3-ppm precursor tolerance and a 10-ppm fragment tolerance.
3. Metabolic Profiling CCS Library search plugin with a 3-ppm precursor tolerance, a 10-ppm fragment tolerance, and a 10% CCS tolerance.

Putative identifications were accepted with a score of >20. In the case that multiple identifications met the criteria, the one with the highest matching score was chosen. In the case that more than one putative identification shared the same score, no identification was retained.

### Data processing and statistical analysis

Data processing and statistical analysis were performed using R version 4.3.1 (2023-06-16) with the ‘StructToolbox’ package [38]. Instrumental drift and batch effects were assessed using pooled QC samples.

Prior to statistical modelling, feature intensities from both chromatographic modes (C18 and HILIC) were normalised using probabilistic quotient normalisation (PQN) referenced to pooled quality control (QC) samples, followed by k-nearest neighbour imputation (k = 5) of missing values and generalised logarithmic (glog) transformation to stabilise variance. Signal drift and batch effects were corrected using QC-based spline/loess batch correction. For the HILIC dataset, residual batch structure was further addressed using ComBat empirical Bayes batch correction. As per standard approaches, feature filtering was applied sequentially: features were removed if present at ≥ 5-fold higher intensity in procedural (column) and extraction blanks relative to samples, if missing in > 90% of samples within a class, or if exhibiting a QC relative standard deviation (RSD) > 40%. While this risks removing very low abundance endogenous metabolites, it is critical to ensure the robusticity of the data. Principal component analysis (PCA) was used throughout for unsupervised quality assessment, outlier detection, and evaluation of the influence of technical (batch, run order) and biological/demographic (site, sex, age, skeletal side) covariates on data structure.

Univariate comparisons of individual metabolite features between consumption-evidence groups were performed using two-sided Wilcoxon rank-sum tests, with significance denoted at conventional threshold (p < 0.05).

For multivariate classification, partial least squares discriminant analysis (PLS-DA) and sparse PLS-DA (sPLS-DA) models were built separately for each chromatographic mode to classify samples by consumption evidence. Samples were partitioned into training (60%) and test (40%) sets using a group-stratified split (grouped by individual, stratified by consumption status) to prevent data leakage between repeated measurements from the same individual. The number of components and the number of variables retained per component were selected via repeated k-fold cross-validation (10-fold, 50 repeats) using the mean classification error rate, and model performance was assessed on the held-out test set using balanced error rate (BER) and confusion matrices. Multi-omic (C18 + HILIC) integration was performed using the DIABLO (Data Integration Analysis for Biomarker Discovery using Latent Components) framework (block sPLS-DA) implemented in mixOmics, with an inter-block design weight of 0.1, and tuned analogously via repeated 10-fold cross-validation.

To assess the influence of demographic and archaeological confounders (sex, site, age, period) on model performance and feature selection, three complementary approaches were used. First, conditional permutation variable importance was computed across the leading ten principal components of each dataset to quantify the contribution of consumption status relative to covariates while accounting for correlation among predictors. Second, metabolite features were residualised against covariates using non-linear random forest regression, and sPLS-DA models were re-fit on the residualised data to evaluate the robustness of feature selection and classification performance (BER, percentage overlap of selected features with the original model). Third, Random Forest classifiers (randomForest, 1000 trees) were trained on metabolomic features alone (Model M1) and on metabolomic features as well as covariates (sex, site, age; Model M3), with performance compared using out-of-bag error, balanced error rate, balanced accuracy, sensitivity, and specificity on the held-out test set, and stability assessed via 10-fold cross-validation (500 trees per fold). Variable importance was quantified as the mean decrease in classification accuracy upon permutation, and partial dependence plots were used to visualise the marginal effect of each covariate on predicted smoking probability.

For individuals lacking direct archaeological evidence of tobacco use, consumption status was predicted using both the sPLS-DA (C18) and Random Forest M3 models. Agreement between the two independent classifiers was quantified as overall percentage concordance and examined stratified by archaeological period (pre-/post-tobacco introduction), site, sex, and age group, with mean RF-predicted class probabilities reported alongside classification frequencies.

All analyses were performed in R, with a fixed random seed (123) applied prior to all stochastic procedures (imputation, batch correction, data partitioning, cross-validation, and model fitting) to ensure reproducibility.

Data analysis is made available at https://github.com/SarahAInskip/THH-Dutch-2026

### Ethical approval

This research was undertaken following ethical guidance outlined by the British Association for Biological Anthropology and Osteoarchaeology. The research was assessed and reviewed by the ethics board at the University of Leicester (ref no 8448-si159-ss/ah:archaeology&ancienthistory). Consent is waived in this case as the individuals are anonymous and we cannot obtain individual consent. However, for the Middenbeemster collection, general consent for research is provided by the local historical society.

## Results

### Study sample and archaeological context

Untargeted LC-MS metabolomic profiling was carried out on skeletal material from three Dutch collections (Arnhem, AR; Middenbeemster, MB; Zwolle, ZW), spanning a Medieval phase (c. 1350–1650, before the European introduction of tobacco) and a Post-medieval phase (c. 1650–1870, after tobacco use became established). Two chromatographic approaches were use to generate data: C18 (reversed-phase, n = 266) and HILIC (n = 197). Skeletal evidence for smoking was recorded as Absent or Present for each individual (Table 1).

### Unsupervised structure and screening for confounding by site, sex, and age

Principal component analysis (PCA) was performed on the full C18 (Fig 1 A, C, E) and HILIC (Fig 1 B, D, F) metabolomic datasets to evaluate whether major sources of biological or archaeological variation were reflected in the overall variance of the data, independent of consumption status. For both assays, the first two components accounted for a modest proportion of total variance (C18: PC1 = 21%, PC2 = 14%; HILIC: PC1 = 21%, PC2 = 12%). With respect to archaeological site (Fig 1 A, B), the C18 dataset showed a modest separation along PC1. This partial clustering indicates that site-associated variation contributes to the overall metabolomic profile in the C18 assay and should be considered a potential confounding factor in downstream group comparisons. In the HILIC dataset, site-related clustering was less pronounced, with AR, MB, and ZW samples showing substantial overlap across both components, suggesting that site exerts a comparatively weaker influence on global metabolomic variance in this assay. No clear separation by sex was observed in either dataset (Fig 1 C, D): female, male, and unknown-sex samples were distributed broadly and homogeneously across PC1 and PC2 in both C18 and HILIC data, indicating that sex is not a major driver of global metabolomic variance and is unlikely to represent a substantial confound for sex-independent analyses. Age-related clustering (Fig 1 E, F) was similarly limited. In the C18 dataset, the youngest age group (20–34) showed a tendency toward positive PC1 values relative to older groups (35–49, 50+), which clustered more centrally and overlapped considerably with one another; the small 20+ (age-indeterminate) group was too sparse to support firm conclusions. The HILIC dataset showed no clear age-related structure, with all age categories broadly co-distributed across the PC1–PC2 space. Univariate analysis with p-values is presented in S1 and S2 Fig and S2 ans S3 Tables.

**Fig 1:**
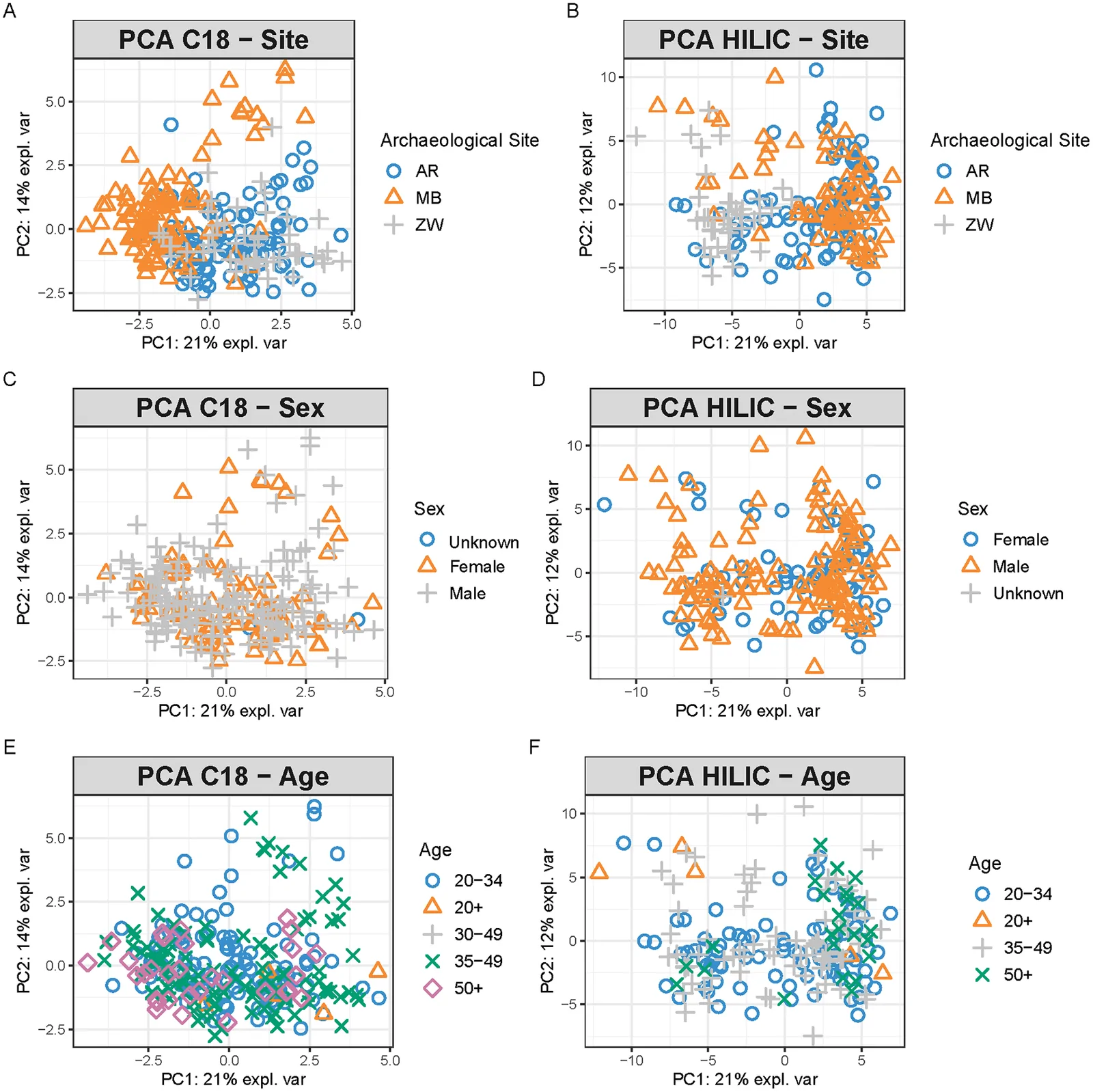
PCA score plots for the C18 (A, C, E) and HILIC (B, D, F) blocks, coloured by recruitment site (A, B), sex (C, D), and age group (E, F).

### Supervised single-block discrimination by sparse partial least squares discriminant analysis (sPLS-DA)

Single-block sPLS-DA was applied to evaluate whether smoking status could be discriminated from the metabolomic profile within each chromatographic block (Fig 2). For the C18 assay, the first two latent variates accounted for 10% and 8% of total variance respectively (Fig 2A). Cross-validated balanced error rate (BER), evaluated across a grid of selected features, ranged from approximately 0.375 for models incorporating four to five components to 0.475 when only the first component was retained (Fig 2B). For the HILIC assay, the first two latent variates explained 13% and 7% of total variance (Fig 2C), with corresponding BER values ranging from approximately 0.38 to 0.43 across the tested feature grid (Fig 2D). In both chromatographic blocks, classification performance exceeded chance expectation, although the magnitude of the observed error rates indicates considerable residual overlap between the smoking-status groups.

**Fig 2:**
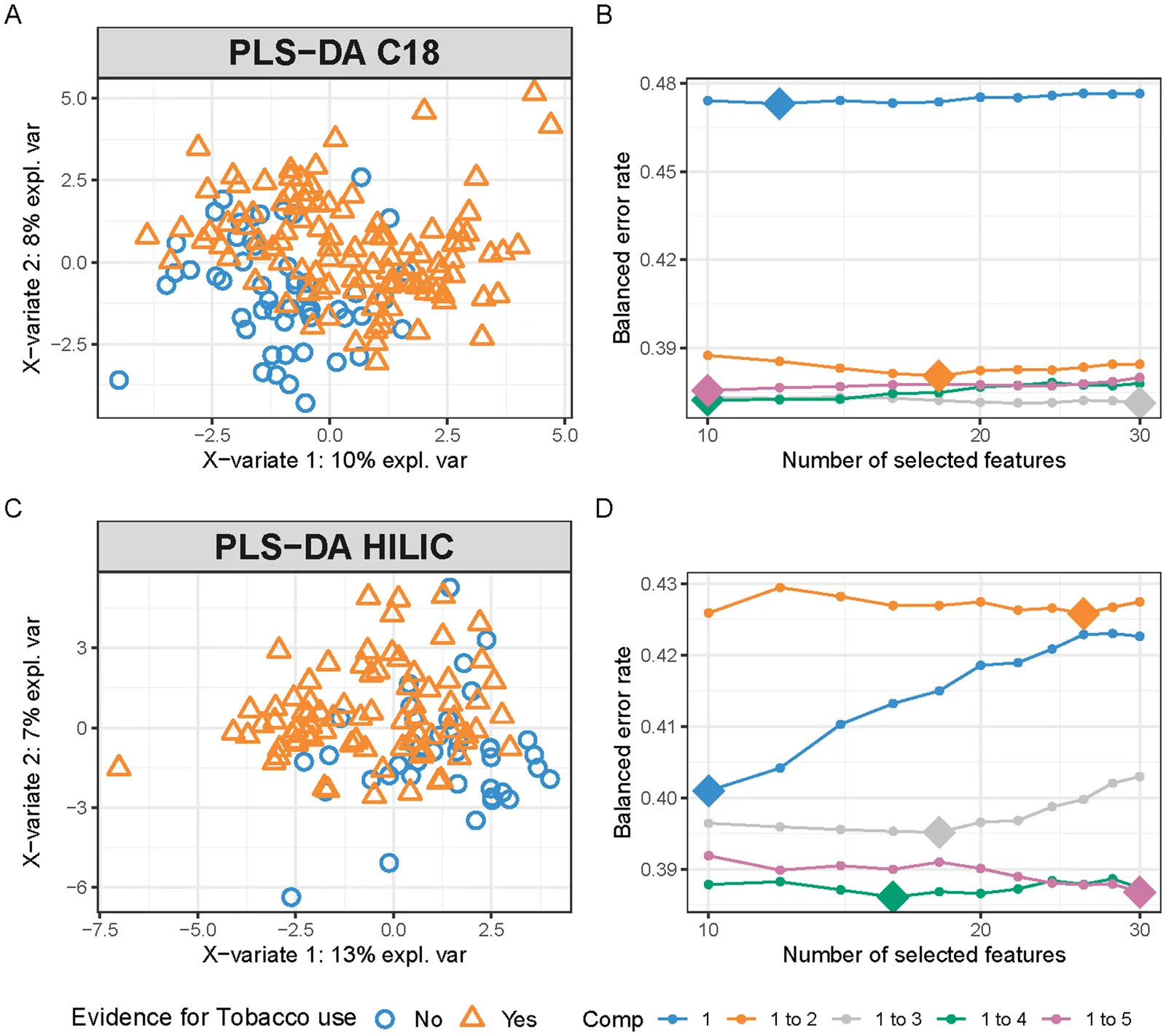
PLS-DA score plots (A, C) and cross-validated balanced error rate vs number of selected features (B, D) for C18 (A, B) and HILIC (C, D).

### Multi-block integration (DIABLO)

A multi-block sparse PLS-DA (DIABLO) model, integrating 30 C18 and 12 HILIC VIP-selected features (42 features total), was fitted jointly across blocks (Fig 3). Sample projections for the first two integrated components showed partial separation of consumption-evidence groups in both blocks (Fig 3A), and loadings for the top contributing features on component 1 of each block showed consistent shifts in standardised values between groups (Fig 3B, C), confirming the univariate and single-block findings. The final DIABLO model cross-validated performance improved with each additional component: using the weighted-vote centroids-distance classifier, the overall error rate dropped from 36.4% at one component to 33.1% at two components and 29.9% at three components, with the balanced error rate (BER) following the same trend (36.2%, 34.0%, and 30.3%). Performance was similar between the two classes at each component, with a discrete improvement for “Yes” (smoking-positive) at three components (29.3% error) compared to “No” (31.3% error). A permutation test based on 10-fold cross-validation (100 permutations) confirmed that the classification performance. The observed classification error rate (CER = 0.284) was significantly lower than expected under random permutation of class labels (p = 0.0198), supporting that the integrated C18/HILIC metabolomic signature carries moderate discriminative information about consumption status rather than reflecting overfitting or coincidental structure in the data. For Metadata for variables selected in the integration model see S5 Table),

**Fig 3:**
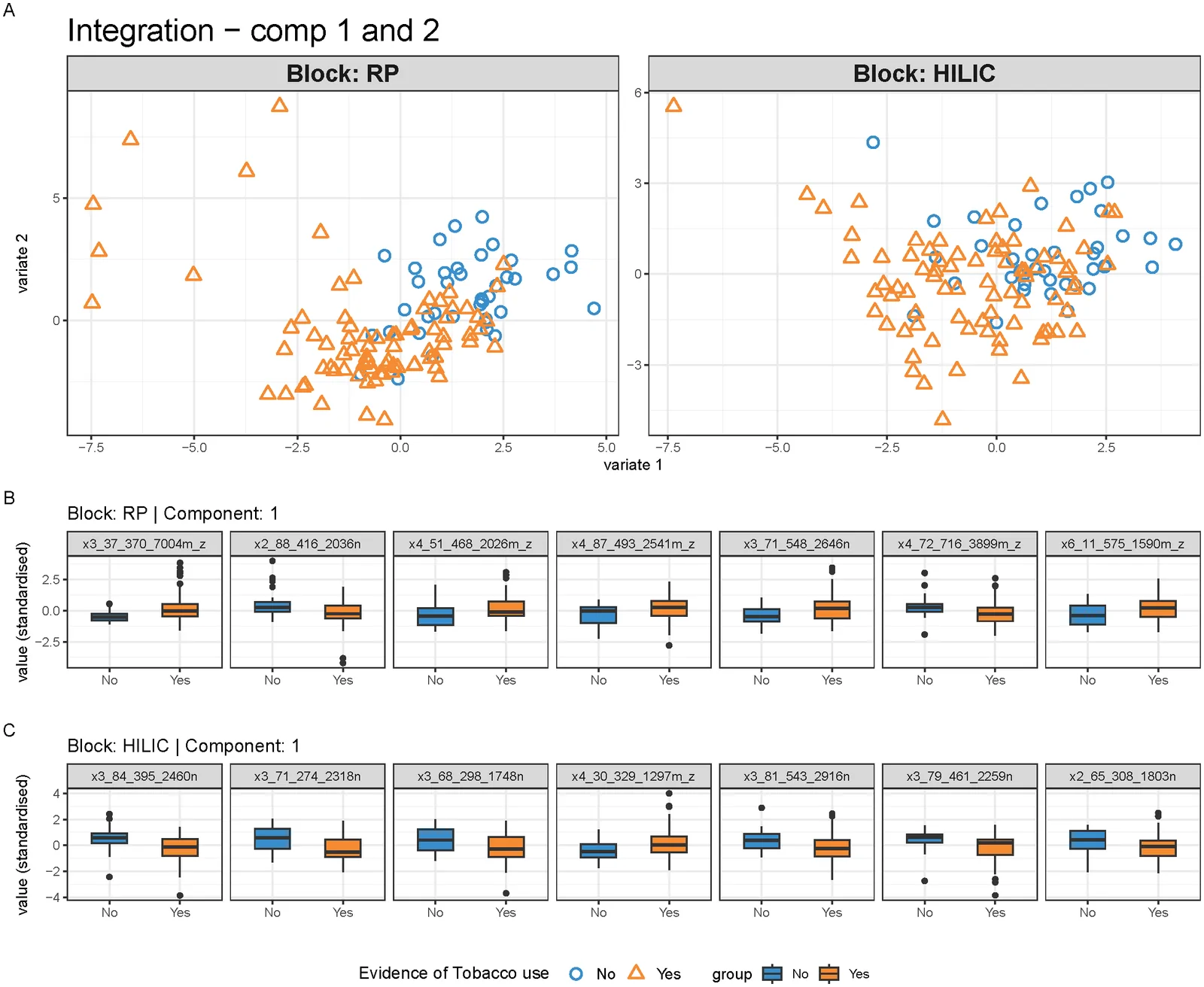
DIABLO integration. (A) Sample projections on integrated components 1 and 2, separately for the RP/C18 and HILIC blocks. (B, C) Standardised values of the top VIP-selected features on component 1, RP/C18 block (B) and HILIC block (C), split by Tobacco use evidence.

### Random forest classification (M1 vs M3) and covariate importance

Random forest classifiers were trained on each block, comparing a metabolomics-only model (M1) with a model additionally including sex, site, and age as covariates (M3). Adding covariates improved both out-of-bag and held-out test performance in both blocks (Table 2), and 10-fold cross-validated BER was correspondingly lower for M3 than M1 (C18: mean BER 0.341 vs 0.445; HILIC: mean BER 0.423 vs 0.528).

**Table 2:** Random forest M1/M3 performance.

Dataset	Model	OOB err.	OOB BER	Test BER	Bal. acc.	Sens.	Spec.
C18	M1	0.304	0.451	0.485	0.515	0.972	0.059
C18	M3	0.261	0.389	0.375	0.625	0.986	0.265
HILIC	M1	0.347	0.435	0.537	0.463	0.811	0.115
HILIC	M3	0.263	0.352	0.489	0.511	0.868	0.154

OOB = out-of-bag; BER = balanced error rate; bal. acc. = balanced accuracy.

In the M3 models, variable-importance rankings showed that, alongside numerous metabolite features, the covariates site and sex ranked among the most important predictors in both blocks, with site showing a markedly higher mean decrease in accuracy than most individual metabolites (Fig 4). Conditional variable importance (which accounts for correlation among predictors) confirmed this. Site had the largest mean conditional importance of any covariate in both blocks, exceeding the archaeological period (pre/post-tobacco) and the consumption-evidence label itself (Table 3). Partial dependence plots for the HILIC M3 model showed higher predicted probability of a positive consumption classification for male and “unknown” sex categories than female, and broadly similar predicted probability across sites and age groups (Figs 5 and 6).

**Table 3:** Conditional (cforest-based) variable importance for site, sex, archaeological period, and consumption-evidence label.

Dataset	Variable	Mean conditional importance
HILIC	site	2.098
HILIC	pre_post_tobacco	0.115
HILIC	evidence_for_consumption	0.030
HILIC	sex_pooled_groups	−0.038
C18	site	2.167
C18	sex_pooled_groups	0.081
C18	pre_post_tobacco	0.057
C18	evidence_for_consumption	0.052

**Fig 4:**
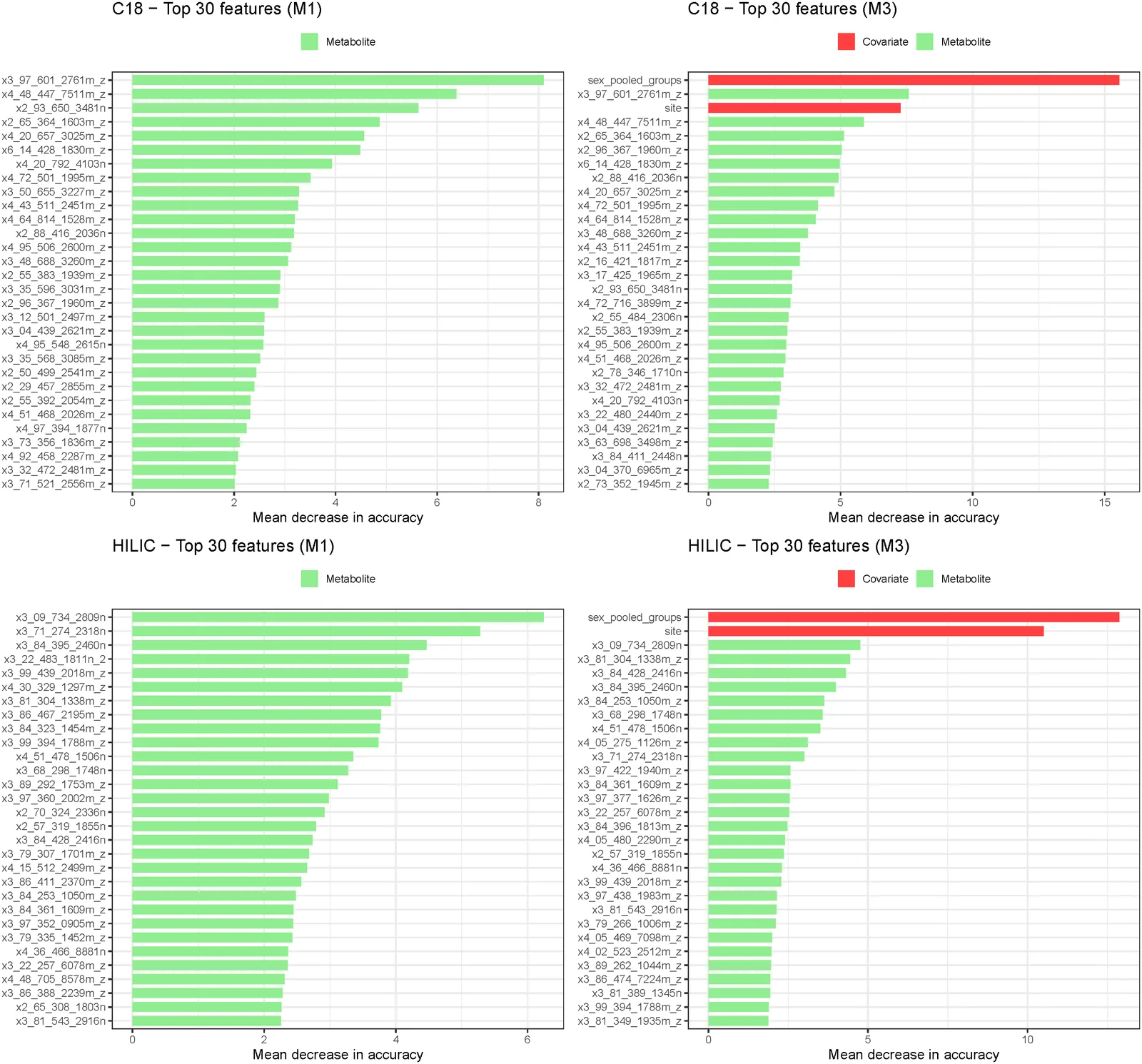
Variable-importance rankings for features in the Random Forest modelling for C18 and HILIC datadata.

**Fig 5:**
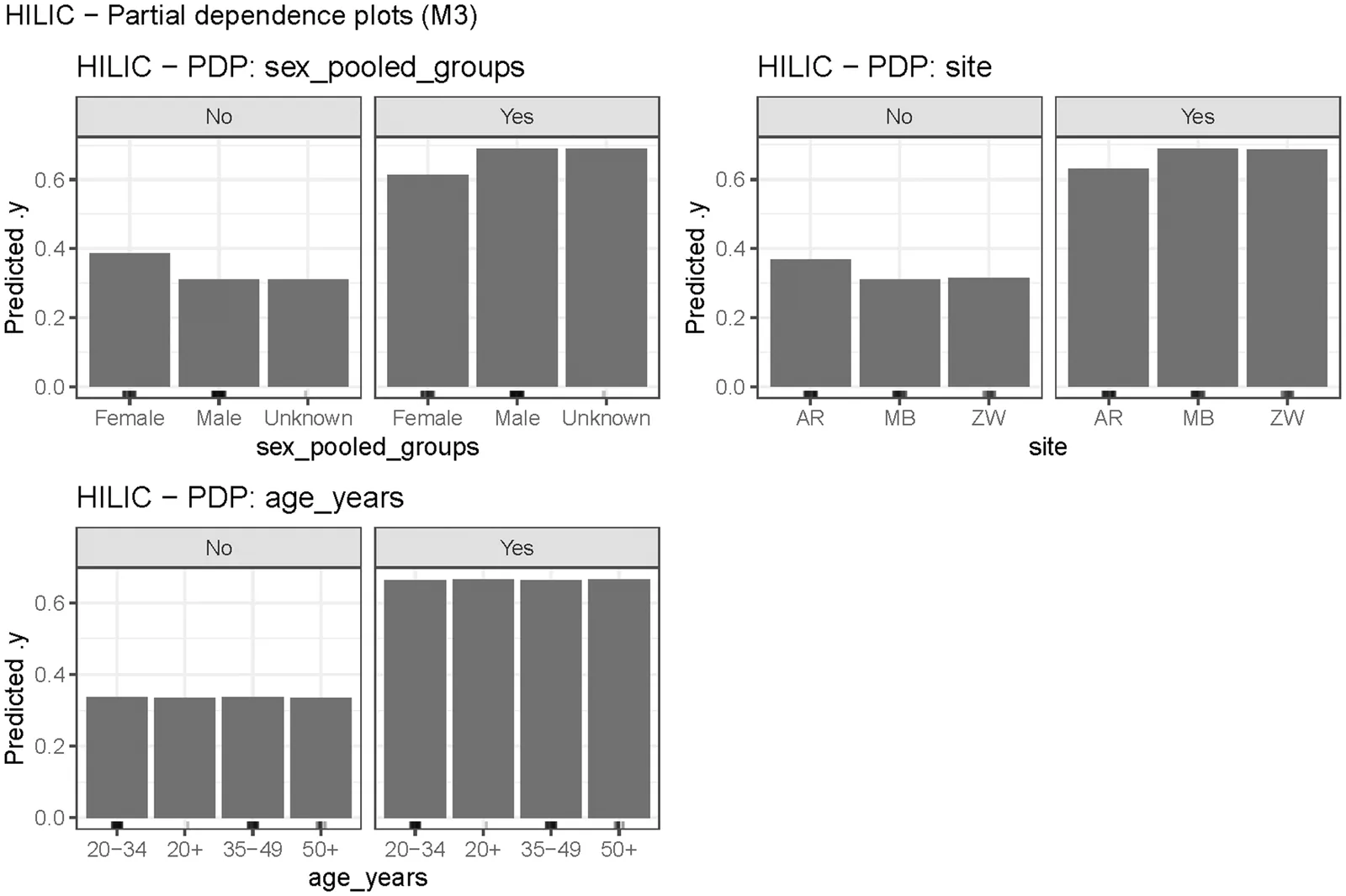
Partial dependence of the HILIC M3 model’s predicted probability on sex, site, and age, shown separately within the “No” and “Yes” evidence-for-pipe notches (irrefutable tobacco consumption indicators).

**Fig 6:**
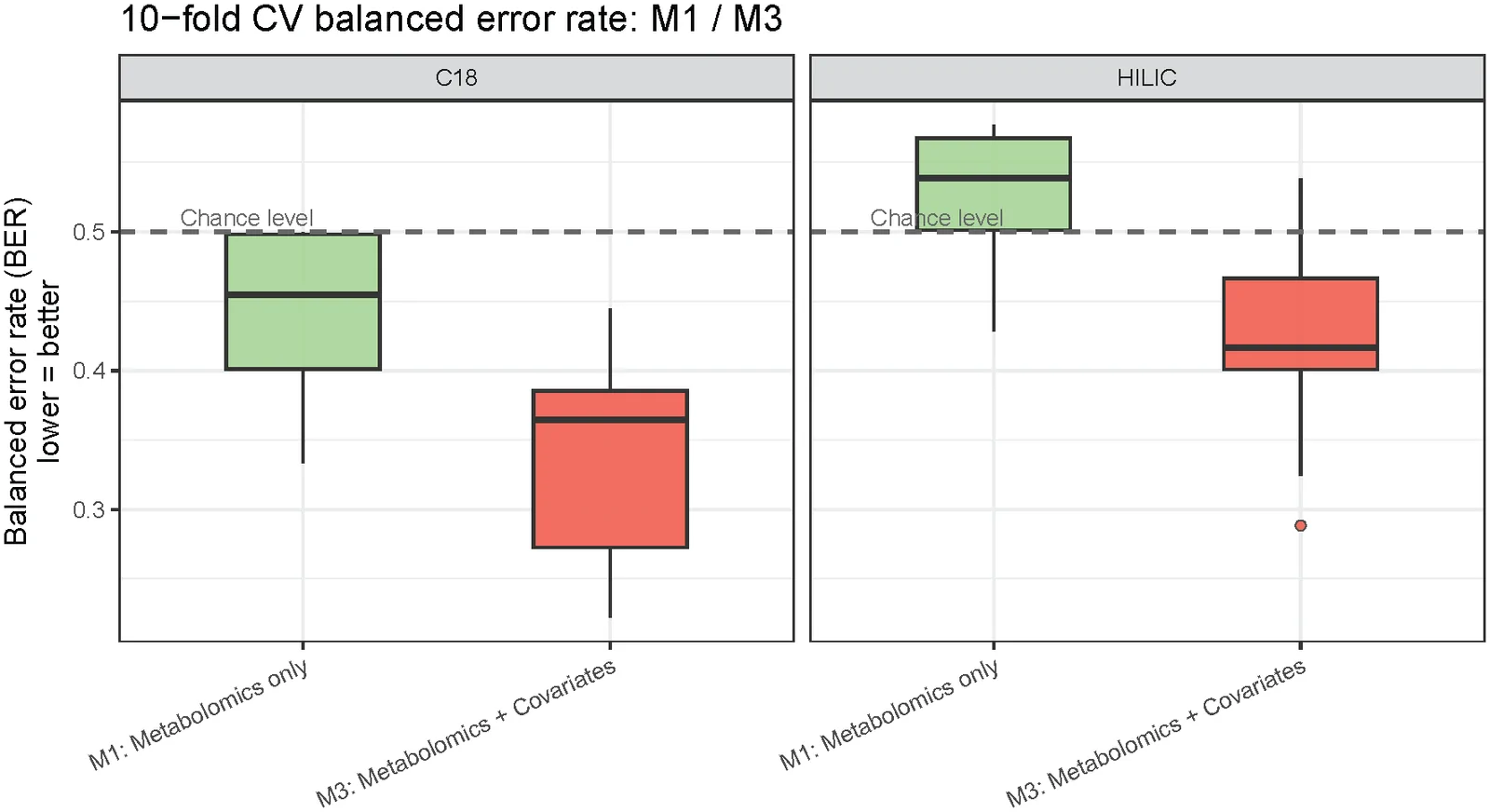
Mean decrease in accuracy for the top 30 features, C18 and HILIC, metabolomics-only ranking vs M3 ranking with covariates highlighted in red.

### Variable importance across all models

The top ten discriminative features identified for each platform and modelling approach are shown in Tables 4 and 5. Consistent with the overlap statistics reported below, several features recur across the RF-M1, RF-M3, and DIABLO VIP panels within each platform. In both assays, sex and collection site (sex_pooled_groups, site) emerged as the top-ranked features in RF-M3, indicating that these covariates carried more importance than any individual metabolite once adjustment was introduced.

**Table 4:** Top 10 features C18 block by model.

C18 – Top 10 Features by Model			
**Rank**	**C18 – RF M1**	**C18 – RF M3**	**C18 – DIABLO VIP**
1	x3_97_601_2761m_z	sex_pooled_groups	x4_43_511_2451m_z
2	x4_48_447_7511m_z	x3_97_601_2761m_z	x3_17_425_1965m_z
3	x2_93_650_3481n	site	x5_80_634_3208m_z
4	x2_65_364_1603m_z	x4_48_447_7511m_z	x4_51_468_2026m_z
5	x4_20_657_3025m_z	x2_65_364_1603m_z	x4_28_565_2642m_z
6	x6_14_428_1830m_z	x2_96_367_1960m_z	x4_46_409_2203m_z
7	x4_20_792_4103n	x6_14_428_1830m_z	x4_87_493_2541m_z
8	x4_72_501_1995m_z	x2_88_416_2036n	x7_71_387_2449m_z
9	x3_50_655_3227m_z	x4_20_657_3025m_z	x4_17_396_1831m_z
10	x4_43_511_2451m_z	x4_72_501_1995m_z	x7_71_379_1024m_z

**Table 5:** Top 10 features HILIC block by model.

HILIC – Top 10 Features by Model			
**Rank**	**HILIC – RF M1**	**HILIC – RF M3**	**HILIC – DIABLO VIP**
1	x3_09_734_2809n	sex_pooled_groups	x3_84_395_2460n
2	x3_71_274_2318n	site	x3_79_541_2834n
3	x3_84_395_2460n	x3_09_734_2809n	x3_79_461_2259n
4	x3_22_483_1811n_2	x3_81_304_1338m_z	x3_81_543_2916n
5	x3_99_439_2018m_z	x3_84_428_2416n	x3_89_413_5232n
6	x4_30_329_1297m_z	x3_84_395_2460n	x3_71_274_2318n
7	x3_81_304_1338m_z	x3_84_253_1050m_z	x3_68_298_1748n
8	x3_86_467_2195m_z	x3_68_298_1748n	x3_63_304_2368n
9	x3_84_323_1454m_z	x4_51_478_1506n	x4_30_329_1297m_z
10	x3_99_394_1788m_z	x4_05_275_1126m_z	x2_86_351_1426n

Overlap between the feature sets selected by each approach was consistently higher for C18 than for HILIC. In C18, 18 of the 30 RF-M1 metabolites were retained among the 28 metabolites of the RF-M3 top-30, 9 of the 30 DIABLO VIP features were also RF-M3 top-30 metabolites, and 15 of the 38 univariately significant features appeared in the RF-M3 top-30, with 7 features common to all three of the univariate, RF-M3, and DIABLO panels. In HILIC, overlap was consistently weaker: 15 of 30 RF-M1 metabolites (50%) were retained in RF-M3, only 5 of 12 DIABLO VIP features were also RF-M3 top-30 metabolites, just 2 of the 16 univariately significant features appeared in the RF-M3 top-30 or the DIABLO VIP panel, and no feature was common to all three HILIC panels.

This pattern indicates that, for C18, the different modelling approaches converge on a partially shared, reproducible panel of discriminative features, whereas for HILIC the approaches largely disagree on which features carry signal consistent with the weaker and less specific univariate and multivariate results already noted for HILIC throughout this section, and suggesting that HILIC-derived feature rankings should be interpreted with more caution than the C18 rankings.

### Comparison of the three modelling approaches

The proportion of individuals classified as consumption-evidence “Yes” differed between the medieval (pre-tobacco) and post-medieval periods across all three approaches, despite none of the models being trained on archaeological period (Table 6). Single-block sPLS-DA classified 34.8% of medieval individuals and 82.0% of post-medieval individuals as “Yes.” Multi-block DIABLO integration classified 27.8% of medieval individuals and 61.2% of post-medieval individuals as “Yes.” Random forest classified 91.3% of medieval individuals and 89.1% of post-medieval individuals as “Yes.”

**Table 6:** Proportion of individuals classified as consumption-evidence “Yes” by archaeological period, across approaches. Gap = % Yes (Post) − % Yes (Pre).

Approach	N (Pre)	% Yes (Pre)	N (Post)	% Yes (Post)	Gap (pp)
sPLS-DA C18, component 1 only	23	34.8%	128	64.1%	+29.3
sPLS-DA C18, components 1–2	23	65.2%	128	82.8%	+17.6
DIABLO integration, component 1	18	27.8%	103	61.2%	+33.4
DIABLO integration, components 1–2	18	38.9%	103	66.0%	+27.1
DIABLO integration, components 1–3	18	44.4%	103	70.9%	+26.5
Random forest, M3	23	91.3%	128	89.1%	−2.2

The multi-block DIABLO integration showed the clearest separation between periods, the single-block C18 sPLS-DA showed an intermediate, smaller gap that shrank further as more components were added, and the random forest M3 model showed essentially no separation at all. It classified medieval, pre-tobacco individuals as consumption-evidence “Yes” at a slightly higher rate (91.3%) than post-medieval individuals (89.1%).

Direct comparison of the C18 sPLS-DA and random forest M3 predictions on the same 151 individuals showed 82.8% concordance (128/151). Where the two methods disagreed, random forest reclassified more sPLS-DA “No” calls to “Yes” (21 individuals) than the reverse (6 individuals reclassified “Yes” to “No”), consistent with random forest higher overall positive rate and its poorer specificity on the period benchmark above.

A complementary residualisation analysis tested this directly by regressing out site, sex, and age from the selected feature panels before re-classifying smoking status (Table 7). For both datasets, cross-validated BER rose sharply toward, or above, chance level after residualization (HILIC: 0.51 to 0.623; C18: 0.368 to 0.623), and only 42–60% of the residualised feature panel overlapped with the original selected features.

**Table 7:** Effect of residualizing feature panels for site, sex, and age before re-classification.

dataset	model	BER	error_rate	n_features	pct_overlap_with_original
HILIC	Original	0.51	0.49	10	100
HILIC	RF-residualized	0.623	0.59	10	50
C18	Original	0.368	0.342	12	100
C18	RF-residualized	0.623	0.6	12	58.33

Considering this together with the conditional-importance results (Table 3), this indicates that a substantial share of the apparent discriminative signal for consumption evidence in the non-residualised models is not necessarily independent from site-, sex-, and age-associated metabolomic variance rather than reflecting consumption-specific chemistry in isolation. Overall, the three approaches converged on a shared set of contributing metabolites (Fig 3B, C and Fig 4) but diverged markedly in apparent specificity once compared against the archaeological tobacco-era transition. The covariate-blind, multi-block DIABLO integration showed the largest and most coherent separation, single-block sPLS-DA was intermediate, and the covariate-including random forest M3 model, despite its lowest cross-validated BER (Table 2), showed no meaningful separation, with site emerging as by far its most influential predictor (Table 3). This pattern suggests that random forest apparent performance advantage in this dataset is driven substantially by site- and sex-associated variance rather than by consumption-specific metabolomic signal, and that classification metrics for M3-type models should be interpreted alongside covariate-importance and specificity checks such as those reported here.

The two independently trained classifiers, a single-block sPLS-DA model and a Random Forest model incorporating covariates were used to estimate consumption status for 151 C18 samples for which no direct pathological/historical evidence of consumption was available (Table 8). Predictions from both models were compared against each samples archaeological period (pre- vs post-medieval, based on pre_post_tobacco), which serves as an independent, albeit indirect and imperfect check: smoking is expected to be far more prevalent in the post-medieval (post-tobacco-introduction) period. Out of 151 unknown samples, each model classified the following numbers (see Table 8) as showing evidence of consumption (Yes) versus not (No).

**Table 8:** Random Forest M3 (C18) predicted consumption status by archaeological period; column percentages.

	sPLS-DA C18 predicted			RF M3 predicted		
	Post	Pre	Total	Post	Pre	Total
No	22 (17.2%)	8 (34.8%)	30 (19.9%)	14 (10.9%)	2 (8.7%)	16 (10.6%)
Yes	106 (82.8%)	15 (65.2%)	121 (80.1%)	114 (89.1%)	21 (91.3%)	135 (89.4%)
Total	128 (100.0%)	23 (100.0%)	151 (100.0%)	128 (100.0%)	23 (100.0%)	151 (100.0%)

The Random Forest M3 model classified a larger proportion of samples as consumption-positive (89.4%) than sPLS-DA (79.5%). The two models agreed on 124 of 151 samples, an overall concordance of 82.1%. Splitting predictions by period shows the expected direction of effect: the percentage classified as consumption-positive is higher in the post-medieval period than the pre-medieval period for both models.

Agreement between the two models is notably weaker in the AR site (70.6% concordant, n = 68), driven mainly by RF M3. Because Arnhem is the only site represented in the pre-medieval group, site and period are confounded for that comparison and should be interpreted with this in mind. Stratifying by sex and age group, sample sizes become small in several cells (n ≤ 5), so the percentages below should be treated as descriptive rather than statistically robust. The remaining site reached a concordance > 90% (Table 9).

**Table 9:** Prediction summary stratified by site.

Site	n	Concordant	sPLS-DA C18 Yes	RF M3 Yes
AR	68	70.6%	57.4%	83.8%
MB	52	98.1%	98.1%	96.2%
ZW	31	90.3%	100%	90.3%

Among the 151 archaeologically unclassified samples with no direct evidence for tobacco consumption, sPLS-DA (C18) and Random Forest (RF M3, metabolomics plus covariates) classifications of consumption status showed high overall agreement (Table 10), particularly in the post-medieval period (See S4 Table for classification). For post-medieval samples (n = 128), the two models agreed on 87.5% of classifications, with sPLS-DA predicting consumption status in 82.8% of individuals and RF M3 predicting consumption in 89.1% (mean RF predicted probability = 0.648). Agreement was lower for the medieval, pre-tobacco-introduction samples (n = 23), at 65.2% concordance; here sPLS-DA classified 65.2% as consumption -positive versus 91.3% for RF M3 (mean RF predicted probability = 0.60), indicating a higher false-positive rate for RF M3 in the period preceding tobacco introduction to the Netherlands.

**Table 10:** Prediction summary stratified by period.

Tobacco consumption	n	Concordant	sPLS-DA C18 Yes	RF M3 Yes
Post	128	87.5%	82.8%	89.1%
Pre	23	65.2%	65.2%	91.3%

Classification concordance between sPLS-DA (C18) and RF M3 varied by sex and age group (Table 11) among the unclassified individuals (See S6 Table for metadata included in analysis).

**Table 11:** Prediction summary stratified by age group and sex.

Sex	Age	n	sPLS-DA_pct_Yes	RF_M3_pct_Yes	pct_concordant
Unknown/Indeterminate	35-49	1	0	100	0
Unknown/Indeterminate	50+	1	100	100	100
Female	20+	4	100	50	50
Female	20-34	18	50	83.3	66.7
Female	35-49	49	85.7	89.8	87.8
Female	50+	43	88.4	88.4	95.3
Male	20-34	5	20	80	40
Male	35-49	14	78.6	100	78.6
Male	50+	16	93.8	100	93.8

Agreement was generally high in older age categories but more variable among younger adults. Among males, concordance increased with age: 40% in the 20–34 group (n = 5), 78.6% in the 35–49 group (n = 14), and 93.8% in the 50 + group (n = 16), with RF M3 predicting smoking in a consistently higher proportion of individuals (70–100%) than sPLS-DA (20–93.8%) across all male age categories. Among females, concordance was 50% in the 20–34 group (n = 4), 66.7% in the 35–49 group (n = 18), and 95.3% in the 50 + group (n = 43), again with RF M3 yielding higher predicted consumption prevalence (83.3–89.8%) than sPLS-DA (50–85.7%). The two individuals of indeterminate sex (35–49 and 50 + , n = 1 each) showed complete disagreement between models (0% concordance), with sPLS-DA predicting no consumption evidence in both cases while RF M3 predicted consumption in both. Overall, RF M3 showed a systematic tendency to classify more individuals as consumption-positive than sPLS-DA across nearly all sex and age strata, and model agreement was highest among individuals aged 50 and above regardless of sex.

Both models classify the majority of unknown individuals as consumption-positive, and both show a higher Yes-rate in the post-medieval period than the pre-medieval period, in a direction consistent with the historical timeline of tobacco use. Random Forest M3 predicts consumption-positive more liberally than sPLS-DA overall, and this difference is greatest in the pre-medieval group, where RF M3 high Yes-rate (91.3%) is more difficult to reconcile with the expected pre-tobacco background. Overall model concordance is 82.1%, but this varies substantially by subgroup. Site and period are confounded in this dataset, since Arnhem is the only site with pre-medieval samples, which limits how independently the effects of site and period on model agreement can be interpreted. Several demographic strata also have very small sample sizes (n ≤ 5), so percentages for these groups should be read as indicative rather than conclusive.

The imbalance in group sizes between the two archaeological periods (post-medieval, n = 128; pre-medieval, n = 23) warrants caution in the interpretation of the period-stratified classification results. With a pre-medieval sample size of only 23, each individual case corresponds to a shift of approximately 4.3% in the reported classification rate, rendering estimates for this group considerably less stable than those derived for the larger post-medieval subset. The apparent Yes-classification rates observed for the pre-medieval group should therefore be interpreted with caution.

This imbalance may also have influenced model behaviour more directly. If the labelled training data used to build the classifiers were themselves skewed toward consumption-positive cases, both models would be expected to exhibit a systematic bias toward predicting the majority class, independent of genuine input signal. Such a mechanism offers a plausible explanation for the near-absence of period-related discrimination observed in the Random Forest M3 model (89.1% post-medieval versus 91.3% pre-medieval), a pattern more consistent with a majority-class default than with a biologically meaningful classification of consumption status. The sPLS-DA model exhibited a larger and more archaeologically plausible difference between periods. However, even this estimate cannot be fully isolated from the sampling variability inherent to a group of only 23 individuals.

This limitation is also linked to the fact that the pre-medieval subset derives exclusively from a single site (Arnhem), introducing a further confound between site and period that cannot be resolved with the present sampling structure. Considering all these aspects, the elevated pre-medieval Yes-classification rate cannot be confidently attributed to a genuine archaeological or biological signal, an artefact of class-imbalanced model training, or the wide confidence intervals associated with small sample sizes, and this ambiguity should be explicitly acknowledged as a limitation when reporting period-based comparisons of predicted consumption status. We discuss this further below.

## Discussion

This study aimed to further evaluate the potential of LC-MS-based metabolomics on archaeological cortical bone to assess tobacco consumption habits in historical populations following successful application on an historic population [4]. This approach is deemed especially important for identifying individuals where there are no macroscopic features of tobacco consumption present in the human skeleton. To achieve this, we employed a combination of C18 and HILIC chromatography, coupled with ion mobility mass spectrometry in MS1 positive acquisition mode to analyse the bone metabolome from archaeological Dutch individuals. Initially, the two assays were modelled separately using sparse Partial Least Squares Discriminant Analysis (sPLS-DA) and then integrated through block sPLS-DA. The results indicated that the C18 assay demonstrated greater stability, while the HILIC assay showed a significant drift during data acquisition (https://github.com/SarahAInskip/THH-Dutch-2026). Despite these differences, both assays achieved similar accuracy in classifying consumption habits, and their integration led to a slight improvement in classification performance. This classification was further validated by comparing unknown samples with their historical context (e.g., pre or post tobacco) confirming the performance of the classification and accounting for the uncertainty of the original classification based on the morphological features. The overall classification proved to be in line with the high rates of pipe-notches seen in the dentition of individuals after this period [16]. While the metabolomic models do produce some high rates of misclassification, we still feel it is a valuable contribution to reconstructing smoking profiles in our past population, especially since we have such clear macroscopic evidence of tobacco use with which we can offset this. However, it does show the importance of using a multimethod approach. The ability to identify and classify individuals as tobacco consumers is particularly valuable to archaeology, but also has significant applications in forensic sciences, where reconstructions of different physical and biological attributes of unknown remains is a priority for individual identification.

As it was identified in PCA that other variables might influence the metabolome and results, we undertook further modelling using Random Forest Analysis M3 to try to account for these factors. Importantly, this identified that there were significant interactions between other co-variables and tobacco consumption status, especially site and potentially sex. Given that soil is a highly complex medium with highly variable composition [39], and that this will have a significant effect on digenesis and decomposition between areas [39,40], it is unsurprising that site had a significant impact on the metabolomic composition of bone. In addition, sex also had some influence as well. It is well known that there are significant differences in the metabolome of males and females, not limited to sex hormones [41], which have been shown to be detectable archaeologically [7]. Other factors may also confound this difference. For example, it is possible that these biological sex differences could be conflated with, for this period, gender-based practices. Based on the macroscopic evidence alone, tobacco consumption appeared to be strongly associated with the male sex for our communities and was also historically documented to be [42]. This led to a significant issue for us in that we had low numbers of non-tobacco using males and definitely tobacco using females to create and test our models, which then made it difficult to know whether differences were truly relating to tobacco consumption or biological variation between the sexes, since most non-tobacco users were female. A possible way forward for this would be to add a further biomolecular method, the detection of tobacco metabolites in preserved dental calculus [14].

Understanding the complexity of classifying tobacco consumption in archaeological skeletal remains requires consideration of the diverse ways in which tobacco was consumed in the past. Tobacco use varied significantly, with people using via pipes, enemas, drinks, cigars, snuffs, and other mediums. Specific physical markers for some of these consumption methods, such as pipe notches or staining on the inner (lingual) surfaces of teeth, can help us identify individuals as definite tobacco consumers. As used here, these features can be used to train models based on the metabolomic profile for classifying unknown individuals. However, such markers are typically associated with pipe or cigar smoking and require well-preserved teeth for assessment. The other mediums of tobacco consumption or even exposure to second-hand smoke could also influence the metabolome but would not leave visible traces on skeletal remains and, therefore, compromise the initial classification and the calculated performance of the models. At present, we do not know whether our model could be affected by these methods of use, especially for those that ingested or inhaled unburnt tobacco (snuff), which would be metabolised in a different body system (digestion). A further limitation of relying on morphological analysis for initial classification is that similar wear patterns could theoretically result from non-tobacco-related activities, although most pipe wear facets are associated with staining. To address this limitation, we incorporated the archaeological context (e.g., pre- or post-tobacco dating) to help validate the metabolomics-based classification, adding an additional layer of complexity to the analysis.

Nonetheless, the classifications presented in Tables 2 and 3 indicate that a substantial proportion of individuals were correctly categorised with respect to tobacco exposure, demonstrating the overall reliability of the analytical approach. However, instances of misclassification were observed, which may be attributable to multiple factors. First, chemical or physical alterations may occur during cleaning or laboratory preparation procedures, potentially affecting the integrity of the samples. This is particularly problematic for already curated skeletal material such as that used here where it has been standard to clean skeletal remains for long-term storage using water. This can lead to the loss of chemical compounds preserved in the bone tissue, potentially compromising the original metabolic signature indicative of tobacco exposure [15,43].

Second, and as discussed further below, inaccuracies in archaeological dating or classification of the archaeological context, as well as the possibility of early or sporadic tobacco use, could also contribute to apparent discrepancies in the data. This is unfortunately an inherent problem in archaeology, although we managed to mitigate this in two of our assemblages where we have known burials with dates of death. Taken together, these factors highlight both the strengths and limitations of using biomolecular markers for reconstructing past patterns of tobacco consumption.

Third, the degradation of endogenous compounds in some individuals through diagenetic processes could reduce the detectability of biomarkers, leading to underestimation of exposure. The degradation of compounds remains a key area for future research, as environmental factors such as hydrolysis, oxidation, and other chemical processes can significantly alter the metabolomic profile [44]. Furthermore, leaching of metabolites through water and other solvents during burial and excavation can complicate the interpretation of metabolomic data. This degradation process may occur both due to environmental agents in the depositional context and during anthropogenic cleaning procedures prior to storage. These factors could explain the presence of non-endogenous compounds in the bone profiles, despite the use of MS1 annotation. Additionally, the metabolomic profile may be subject to contamination from soil, water, and other organic materials, which can vary significantly across different archaeological sites. Such site-specific contamination is a limitation when attempting to identify universal markers for tobacco exposure. Our findings, supported by principal component analysis (PCA) and the Random Forest modelling, suggest that metabolomic profiles differ according to the archaeological context, indicating the influence of site-specific factors. Moreover, differential preservation conditions across sites could lead to variable loss of key metabolites, affecting the overall results. However, isolating individual archaeological sites would drastically reduce sample size for the current study and, as a result, reduce statistical robusticity. Further studies should focus on the inter-site variation on large representative samples.

Forth, the nature of bone tissue, which is characterised by low metabolic activity, and the long deposition times involved, likely exacerbate this issue. As observed in Fig 5B-C, the expected biological variation linked to tobacco consumption was not as clear-cut as anticipated. Alldritt et al [11] identified that plant derived secondary metabolites can become sequestered in cells after consumption, and that some of these molecules bind to bone (hydroxyapatite), some of them strongly so, and may end up there during the remodelling process. First, it is possible that some individuals were infrequent users of tobacco, or that they ceased using tobacco for longer than they used it and perhaps did not accumulate many associated metabolites. It was observed here that classification concordance between our models improved in the older age categories. As bone remodelling is relatively slow and retains structure from long time ranges [45], it is possible that older individuals, whose bones have had a far longer time to accumulate metabolites within their osseous structures, might be easier to classify. Further work is needed to understand the relationship between positive tobacco consumption classification and duration and frequency of tobacco use along with the mechanisms of sequestering of exogenous metabolites in bone.

Finally, without detailed historical records about burials (e.g., burial registers and records) and/or intensive radiocarbon dating, site dating can often be vague. This is an inherent problem in archaeology. In addition, the exact date that tobacco became popular in the Netherlands is also debated with new work suggesting it was potentially earlier than previously thought [16,46]. It became apparent after our sampling and analysis that some of the skeletons from the pre-tobacco use phase groups had pipe-notches suggesting that some of the apparently misclassified pre-tobacco non-exposed control individuals might have used or been exposed to tobacco. As such, it is possible that some individuals were in fact not misclassified but in fact retrospectively ended up in the wrong control group. Issues of second-hand smoke exposure would also be a problem here too. Like the analysis of material from England [4], which had lower misclassification and where we avoided individuals that could come from very early tobacco contexts, future work needs to have more securely dated material to construct models to avoid this issue. As such, Bayesian modelling using C14 dating and other lines of evidence need to be built into experimental designs.

## Conclusions

We provided a robust biochemical and statistical approach for the detection of differences in the bone metabolome between tobacco users and non-users in an archaeological population from the Netherlands. We also highlighted several limitations that emerged from the current untargeted metabolomic studies on archaeological bone (database limitations, sample composition and availability, archaeological dating, lack of knowledge on (variable) impact of digenesis). We would like to stress the importance of considering all archaeological data when developing classification models based on biochemical or molecular characteristics of the remains. Comparing classification results with site chronology is, in this case, the preferred choice for this dataset but this information should be included in the models in future studies involving a more comprehensive and balanced sample. Furthermore, where possible, systematic methods for dating archaeological material need to be incorporated into metabolomic studies where time period is a key variable. We identified molecules that would be valuable for further research into the identification of biomarkers for detecting past tobacco exposure in bone. Finally, it is imperative that further work is undertaken on additional populations under different taphonomic conditions to help disentangle the impact that these variables have on the bone metabolome and the stability of any biomarkers identified.

## Supporting information

S1 Text Information about the sites used in the research. (DOCX)

S1 Table Table outlining the number of samples and the individuals they pertained to in the Dutch collections. (XLSX)

S2 Table Features metadata for significantly associated with smoking status, identified using the Wilcoxon rank-sum test with C18. Only metabolites with p-values indicating statistical significance are reported.
(XLSX)

S3 Table Features metadata for significantly associated with smoking status, identified using the Wilcoxon rank-sum test with HILIC. Only metabolites with p-values indicating statistical significance are reported.
(XLSX)

S4 Table Classification of the complete unknown sample set using the sPLS-DA model developed from the C18 dataset, which represents the most comprehensive dataset. (XLSX)

S5 Table Metadata for variables selected in the integration model, as well as for the individual HILIC and C18 single-block models, detailing the features included in each analysis and their selection criteria. (XLSX)

S6 Table Skeletal metadata and results of the concordance between the sPLS-DA and RF modelling. (XLSX)

S1 Fig Boxplots illustrating the relative intensity of C18 features significantly associated with smoking status, as determined by the Wilcoxon rank-sum test. (PDF)

S2 Fig Boxplots illustrating the relative intensity of HILIC features significantly associated with smoking status, as determined by the Wilcoxon rank-sum test. (PDF)
