# Supplementary material for: Using archaeometabolomics to characterise tobacco-usage in human cortical bone from 17th to 19th century Dutch communities
Source: PLoS One. 2026 Oct 30;21(10):e0361147. doi: 10.1371/journal.pone.0361147 (PMC13722171; doi:10.1371/journal.pone.0361147)
Supplement: Supplementary file 1 [file pone.0361147.s008.pdf]

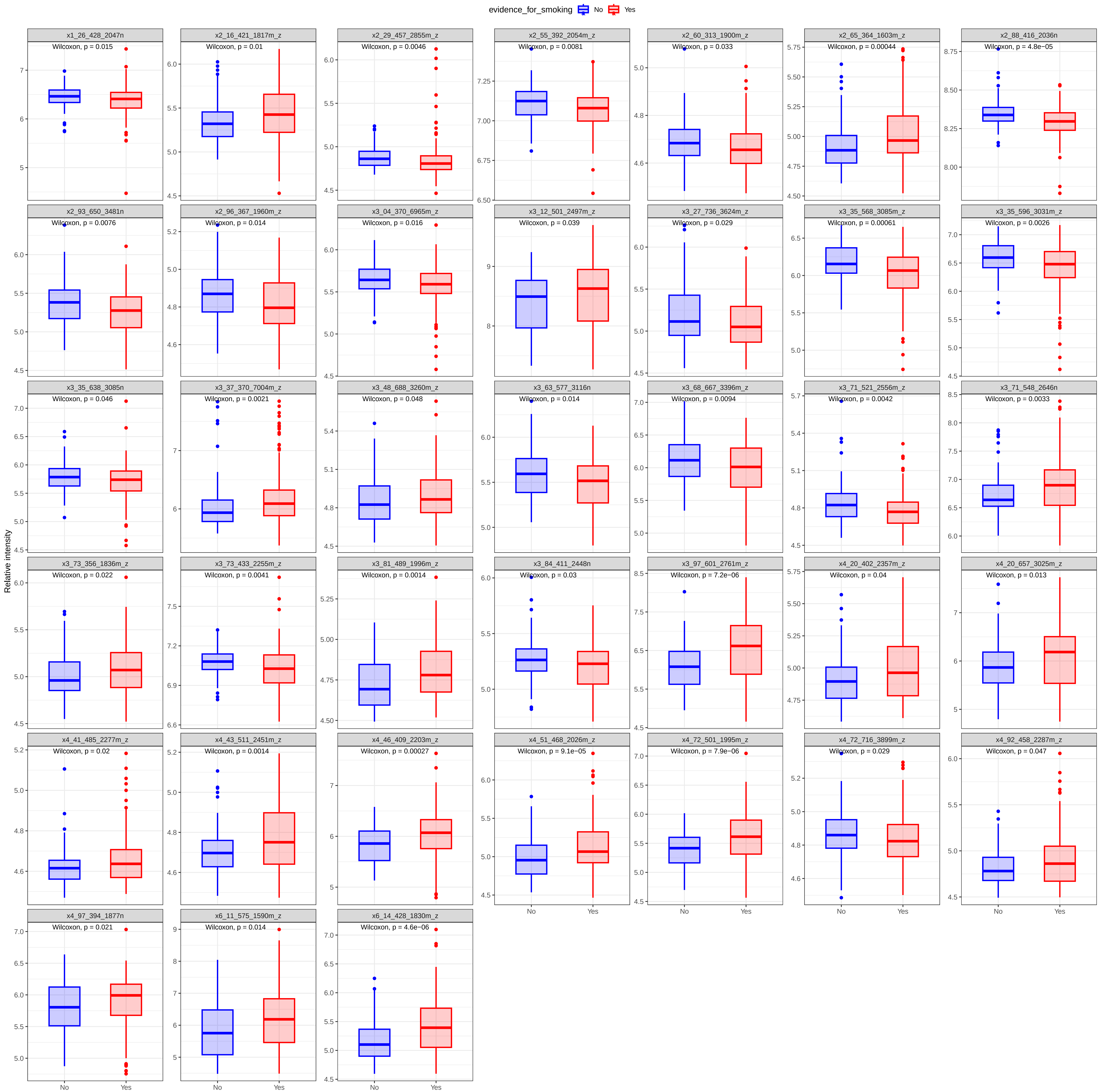
evidence_for_smoking No Yes
Relative intensity
x1_26_428_2047n Wilcoxon, p = 0.015
x2_16_421_1817m_z Wilcoxon, p = 0.01
x2_29_457_2855m_z Wilcoxon, p = 0.0046
x2_55_392_2054m_z Wilcoxon, p = 0.0081
x2_60_313_1900m_z Wilcoxon, p = 0.033
x2_65_364_1603m_z Wilcoxon, p = 0.00044
x2_88_416_2036n Wilcoxon, p = 4.8e−05
x2_93_650_3481n Wilcoxon, p = 0.0076
x2_96_367_1960m_z Wilcoxon, p = 0.014
x3_04_370_6965m_z Wilcoxon, p = 0.016
x3_12_501_2497m_z Wilcoxon, p = 0.039
x3_27_736_3624m_z Wilcoxon, p = 0.029
x3_35_568_3085m_z Wilcoxon, p = 0.00061
x3_35_596_3031m_z Wilcoxon, p = 0.0026
x3_35_638_3085n Wilcoxon, p = 0.046
x3_37_370_7004m_z Wilcoxon, p = 0.0021
x3_48_688_3260m_z Wilcoxon, p = 0.048
x3_63_577_3116n Wilcoxon, p = 0.014
x3_68_667_3396m_z Wilcoxon, p = 0.0094
x3_71_521_2556m_z Wilcoxon, p = 0.0042
x3_71_548_2646n Wilcoxon, p = 0.0033
x3_73_356_1836m_z Wilcoxon, p = 0.022
x3_73_433_2255m_z Wilcoxon, p = 0.0041
x3_81_489_1996m_z Wilcoxon, p = 0.0014
x3_84_411_2448n Wilcoxon, p = 0.03
x3_97_601_2761m_z Wilcoxon, p = 7.2e−06
x4_20_402_2357m_z Wilcoxon, p = 0.04
x4_20_657_3025m_z Wilcoxon, p = 0.013
x4_41_485_2277m_z Wilcoxon, p = 0.02
x4_43_511_2451m_z Wilcoxon, p = 0.0014
x4_46_409_2203m_z Wilcoxon, p = 0.00027
x4_51_468_2026m_z Wilcoxon, p = 9.1e−05
x4_72_501_1995m_z Wilcoxon, p = 7.9e−06
x4_72_716_3899m_z Wilcoxon, p = 0.029
x4_92_458_2287m_z Wilcoxon, p = 0.047
x4_97_394_1877n Wilcoxon, p = 0.021
x6_11_575_1590m_z Wilcoxon, p = 0.014
x6_14_428_1830m_z Wilcoxon, p = 4.6e−06
No Yes
