# Supplementary material for: Using archaeometabolomics to characterise tobacco-usage in human cortical bone from 17th to 19th century Dutch communities
Source: PLoS One. 2026 Oct 30;21(10):e0361147. doi: 10.1371/journal.pone.0361147 (PMC13722171; doi:10.1371/journal.pone.0361147)
Supplement: Supplementary file 2 [file pone.0361147.s009.pdf]

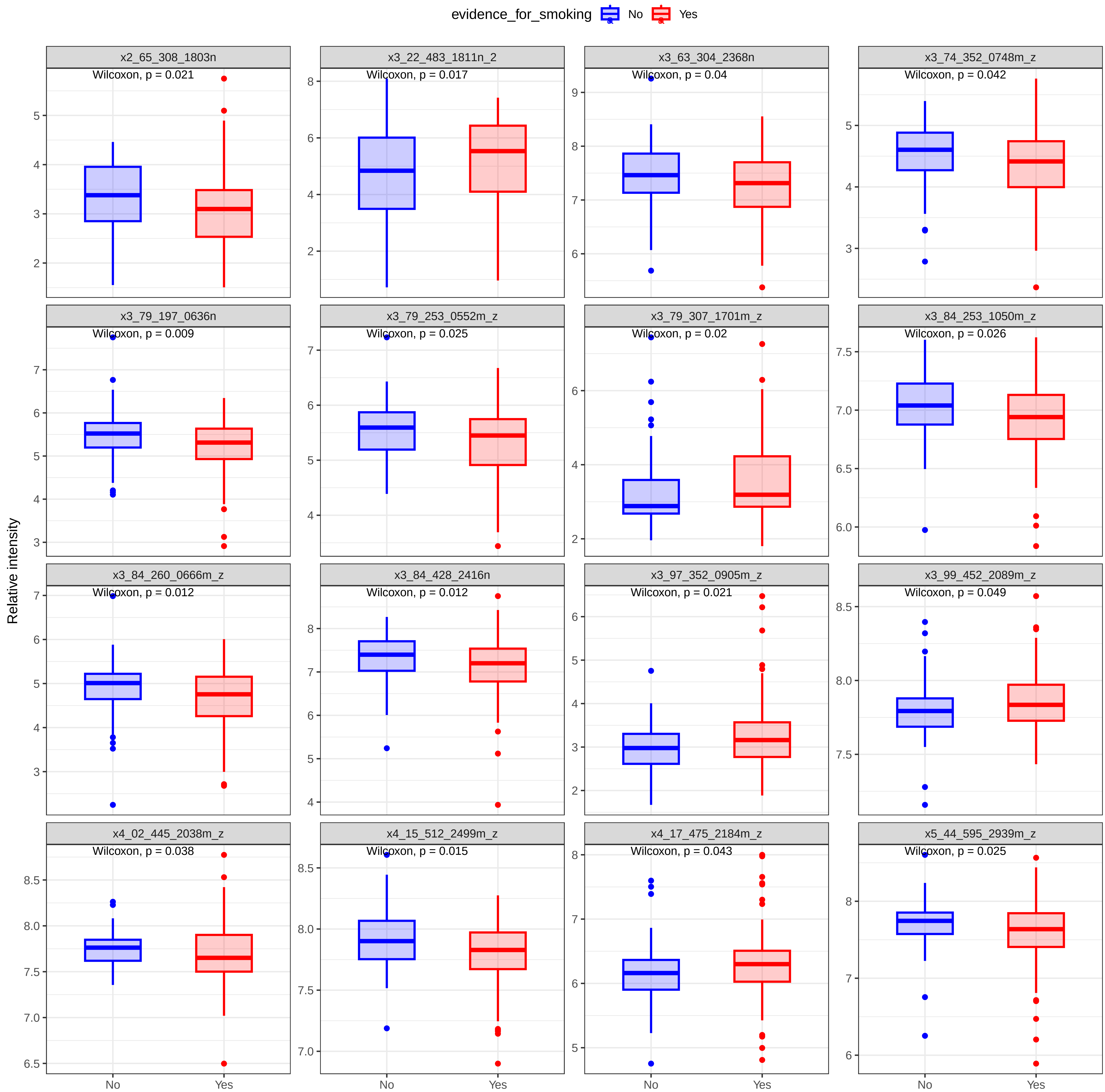

evidence_for_smoking No Yes
Relative intensity
x2_65_308_1803n
Wilcoxon, p = 0.021
x3_22_483_1811n_2
Wilcoxon, p = 0.017
x3_63_304_2368n
Wilcoxon, p = 0.04
x3_74_352_0748m_z
Wilcoxon, p = 0.042
x3_79_197_0636n
Wilcoxon, p = 0.009
x3_79_253_0552m_z
Wilcoxon, p = 0.025
x3_79_307_1701m_z
Wilcoxon, p = 0.02
x3_84_253_1050m_z
Wilcoxon, p = 0.026
x3_84_260_0666m_z
Wilcoxon, p = 0.012
x3_84_428_2416n
Wilcoxon, p = 0.012
x3_97_352_0905m_z
Wilcoxon, p = 0.021
x3_99_452_2089m_z
Wilcoxon, p = 0.049
x4_02_445_2038m_z
Wilcoxon, p = 0.038
x4_15_512_2499m_z
Wilcoxon, p = 0.015
x4_17_475_2184m_z
Wilcoxon, p = 0.043
x5_44_595_2939m_z
Wilcoxon, p = 0.025
No
Yes
