# Supplementary material for: Using archaeometabolomics to characterise tobacco-usage in human cortical bone from 17th to 19th century Dutch communities
Source: PLoS One. 2026 Oct 30;21(10):e0361147. doi: 10.1371/journal.pone.0361147 (PMC13722171; doi:10.1371/journal.pone.0361147)
Supplement: Supplementary file 3 [file pone.0361147.s001.docx]

**Supplementary information**

**Supplementary text 1**

**Site details**

**Middenbeemster**

In 2011, excavations at the Keyserkerk in Middenbeemster revealed a large cemetery containing the remains of over 400 individuals. This cemetery served the entire municipality of Beemster (72 km^2^ region), a rural community mainly consisting of dairy farmers and artisans. Individuals were a mixture of working classes and some more well-off families. The majority of individuals were local or native to the Netherlands, with a few people coming from Germany. The skeletons were mostly very well preserved and were highly complete, with some family plots uncovered. Burials from this cemetery date from approximately AD1638 to 1866, of which most are known to date to between AD1829 and 1866 due to burial records (10). As such, they mostly represent individuals who lived in a time when tobacco was available and was popular. Much research has taken place on these individuals, and this has shown that there was a high rate of pipe notches in the men (31, 32) showing that tobacco use was common place. For this research, a sample of 100 adult individuals were sampled for analysis. The remains are currently stored at the Laboratory for Human Osteoarchaeology at the Faculty of Archaeology at Leiden University, in the Netherlands.

**Broerenkerk in Zwolle, Overijssel, The Netherlands.**

Restructuring works at the Broerenkerk in Zwolle in the 1980s led to the excavation of multiple graves inside the church in 1987-1988 (33). Over 500 individuals were excavated, with 141 of these having demographic information about age and or sex from burial records. It is these latter individuals that were analysed here which are now stored in the Laboratory for Human Osteoarchaeology at the Faculty of Archaeology at Leiden University, in the Netherlands. The burials from Zwolle date from between c. AD1675 and 1828, almost all of those included within the sample falling between AD1819 and 1828 (identifiable via burial records), and consisting of well-to-do middle-class individuals from the city, who were affluent enough to be buried in the desirable position under the floor of the church (7). The burials were highly complete and the preservation of the material is excellent. Previous research on the collection highlighted individuals that had multiple pipe notches and tobacco staining (33). At the time of research, the remains were stored at the Laboratory for Human Osteoarchaeology at the Faculty of Archaeology at Leiden University, in the Netherlands.

**Eusebiuskerk, Arnham, The Netherlands.**

In 2017, large scale renovations in the city of Arnham required excavations at the church Eusebiuskerk. Just over 650 individuals representing men, women and children were excavated from primary graves (e.g. not disturbed) across the site. At the time of research, these could be split into individuals dating from 1350-1650 (mostly before tobacco was popular) and 1650-1829 (once tobacco had become an established popular commodity). For this research, a total of 107 individuals from the church cemetery were sampled. Forty-eight individuals were sampled from the material dating approximately from AD1350-1650, and 59 individuals were sampled from the individuals dating to AD1650-1829. The preservation and completeness of the sampled individuals was very good. In contrast to Zwolle, individuals from the churchyard of Eusebiuskerk are tentatively concluded to have been of average or low socio-economic status (8, 9). They would have made a living in a variety of different ways. Like the other collections, pipe notches were a common finding in the material with 15% of those with teeth having at least one well defined notch (9). At the time of research, the remains were stored at the Laboratory for Human Osteoarchaeology at the Faculty of Archaeology at Leiden University, in the Netherlands.
